# Integrated Approaches for the Use of Large Datasets to Identify Rational Therapies for the Treatment of Lung Cancers

**DOI:** 10.3390/cancers11020239

**Published:** 2019-02-19

**Authors:** Robert J. Cardnell, Lauren Averett Byers, Jing Wang

**Affiliations:** 1 Department of Thoracic/Head and Neck Medical Oncology, The University of Texas MD Anderson Cancer Center, Houston, TX 77030, USA; rcardnell@mdanderson.org (R.J.C.); lbyers@mdanderson.org (L.A.B.); 2 Department of Bioinformatics and Computational Biology, The University of Texas MD Anderson Cancer Center, Houston, TX 77030, USA

**Keywords:** bioinformatics, integrated approaches, lung cancer, rational therapy

## Abstract

The benefit and burden of contemporary techniques for the molecular characterization of samples is the vast amount of data generated. In the era of “big data”, it has become imperative that we develop multi-disciplinary teams combining scientists, clinicians, and data analysts. In this review, we discuss a number of approaches developed by our University of Texas MD Anderson Lung Cancer Multidisciplinary Program to process and utilize such large datasets with the goal of identifying rational therapeutic options for biomarker-driven patient subsets. Large integrated datasets such as the The Cancer Genome Atlas (TCGA) for patient samples and the Cancer Cell Line Encyclopedia (CCLE) for tumor derived cell lines include genomic, transcriptomic, methylation, miRNA, and proteomic profiling alongside clinical data. To best use these datasets to address urgent questions such as whether we can define molecular subtypes of disease with specific therapeutic vulnerabilities, to quantify states such as epithelial-to-mesenchymal transition that are associated with resistance to treatment, or to identify potential therapeutic agents in models of cancer that are resistant to standard treatments required the development of tools for systematic, unbiased high-throughput analysis. Together, such tools, used in a multi-disciplinary environment, can be leveraged to identify novel treatments for molecularly defined subsets of cancer patients, which can be easily and rapidly translated from benchtop to bedside.

## 1. Introduction

While many targeted therapies have been tested in lung cancers, the challenge remains to identify the subset(s) of patients who will respond to these treatments. Integrated approaches are necessary to combine in vitro, in vivo, in silico, and clinical data to identify and validate potential treatments and the cohorts of patients in which these should be used. The University of Texas MD Anderson Lung Cancer Multidisciplinary Program integrates a team of investigators with expertise in biologic, bioinformatics, and clinical studies and has a track record of utilizing high-throughput “-omics” data to identify new therapeutic targets and biomarkers. Here, we discuss three approaches to utilize large datasets with the goal of identifying rational therapeutic options for biomarker-driven patient subsets.

## 2. Datasets

The approaches described in this manuscript use a combination of publically available clinical datasets accessible through cBioPortal for Cancer Genomics (http://www.cbioportal.org/) or the National Center for Biotechnology Information (NCBI) (https://www.ncbi.nlm.nih.gov/), and cell line data from the Broad Institute Cancer Cell Line Encyclopedia (CCLE) (https://portals.broadinstitute.org/ccle), the Genomics of Drug Sensitivity in Cancer (GDSC) (https://www.cancerrxgene.org/), and the National Cancer Institute (NCI) Developmental Therapeutics Program (http://sclccelllines.cancer.gov) summarized in Table 1, in additional to data generated at the University of Texas MD Anderson Cancer Center as part of our cell line characterization efforts and clinical trials. The datasets contain a variety of profiling data including genomic, transcriptomic (either array based or RNASeq), methylation, miRNA expression, and protein expression (by reverse phase protein array (RPPA)), as well as drug response for the cell lines and clinical data for patients (e.g., overall survival, progression free survival, smoking history). Compatibility between datasets is crucial and requires taking into account possible batch effects, probe selection in the use of array-based data [1], and fundamental differences in technology (i.e., microarrays versus RNAseq). For example, when comparing cell line drug sensitivity, the manner in which both single agent [2] and combination data [3,4,5,6] were generated (e.g., length of experiment, drug dose, and dilution factors) must also be carefully considered, to ensure that the approaches used are compatible, and that the analysis model chosen is adequately supported by the data. Sample source must also be considered, regardless of the type of data available. While cell lines can be used for drug sensitivity assays and candidate biomarker discovery, they do not reflect the role of the tumor stroma and immune microenvironment. Patient derived data, however, do reflect the tumor stroma and immune microenvironment and often include outcome data and other clinical parameters, but do not allow for screening of candidate agents. These considerations highlight some of the underlying reasons behind the assembly of the Multidisciplinary Program, drawing upon a range of expertise from across the institution.

## 3. Approaches

### 3.1. Cancer EMT Signature

The concept of epithelial-to-mesenchymal transition (EMT), a process by which epithelial cells lose cellular polarity and cell–cell adhesion and enter a mesenchymal state with enhanced migratory and invasive properties, was first described more than a decade ago in cancer [15,16].

**Figure 1:**
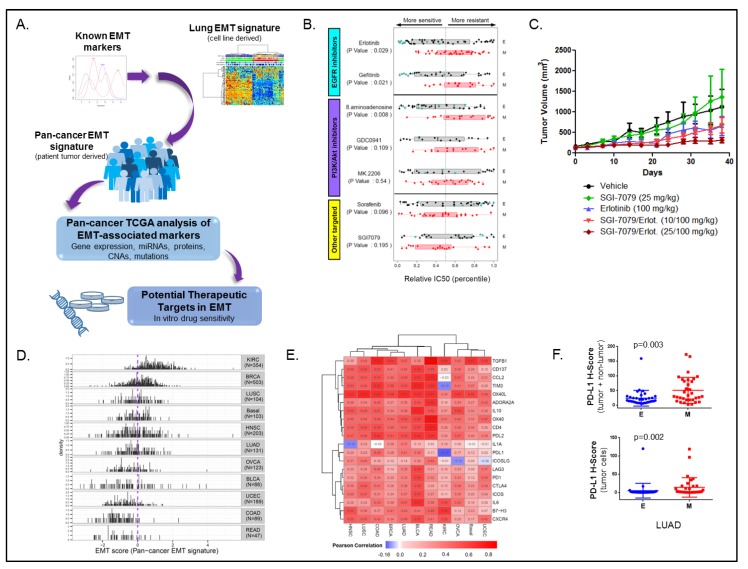
Development of an epithelial-to-mesenchymal transition (EMT) signature. Schematic describing the development of the lung-cancer and pan-cancer EMT scores (**A**). Using the lung-EMT score, mesenchymal cells are less sensitive to EGFR and PI3K inhibition, but are more sensitive to AXL inhibition (**B**). AXL blockade inhibits growth of mesenchymal (A549) non-small cell lung cancer (NSCLC) xenografts (**C**). The Cancer Genome Atlas (TCGA) pan-cancer tumor types display a range of EMT scores (**D**). A mesenchymal pan-cancer EMT score is correlated with higher expression of immune checkpoint genes across multiple cancer types (**E**). Mesenchymal lung adenocarcinoma (LUAD) has higher expression of PD-L1 in both tumor and non-tumor cells by immunohistochemistry (**F**). Adapted from Byers et al. 2013 [17] and Mak et al. 2015 [19].

As EMT plays a role in resistance to standard treatments for non-small cell lung cancer (NSCLC) (and other cancers), and no standard method existed to quantify the degree to which a tumor had undergone EMT, we first developed a lung cancer-specific EMT signature, and subsequently a pan-cancer derived signature based on transcriptomic profiling (Figure 1A) [17]. Using gene expression in 54 NSCLC cell lines, the lung cancer EMT signature was first based on those genes whose mRNA expression levels were significantly correlated (either positively or negatively) with at least one of four putative EMT markers—E-cadherin, vimentin, N-cadherin, and/or fibronectin 1. These “seed genes” were selected as they had previously been established as markers of EMT in lung cancers and other epithelial tumor types. Second, the set of genes correlated to the EMT markers was further limited to those with a bimodal expression pattern to facilitate the ability of the signature to dichotomize the cell lines into distinct epithelial and mesenchymal groups. Third, genes correlated to the EMT markers also had to correlate in an independent mRNA microarray dataset to reduce artifacts and identify the most biologically and technically robust genes. We then used the epithelial or mesenchymal status of the cell lines to determine if EMT predicted response to various targeted agents (Figure 1B). As expected, EGFR inhibitors had greater activity in epithelial models. An interesting observation was that the AXL inhibitor SGI7079 was more efficacious in the mesenchymal models. Expression of AXL, a receptor-tyrosine kinase, was higher in the mesenchymal cell lines, suggesting AXL as a novel target in mesenchymal NSCLC. We then tested the efficacy of SGI7079 in an epithelial mouse xenograft model, where we observed single agent activity and a greater than additive effect when combined with erlotinib (Figure 1C). As a clinical validation of our observations, we classified NSCLC patients with prior systemic therapy and subsequent relapse enrolled to the BATTLE-1 (Biomarker-integrated Approaches of Targeted Therapy for Lung Cancer Elimination) [18] clinical trial as either epithelial or mesenchymal. As expected, *EGFR* wild-type patients with an epithelial tumor treated on the erlotinib arm had significantly better eight-week disease control than those with mesenchymal tumors.

To account for the contribution of the tumor microenvironment to EMT, we built on the lung cell line EMT score, to develop a pan-cancer, patient tumor-derived, EMT score [19]. Using an approach similar to the lung-EMT score, we identified mRNAs best correlated with established “seed” markers of EMT (E-cadherin, vimentin, fibronectin, and N-cadherin) across nine distinct, primarily epithelial, solid tumor types from The Cancer Genome Atlas (TCGA) [9]. Using this approach, we identified 77 genes across the nine tumor types tested (breast invasive carcinoma—BRCA, lung squamous cell carcinoma—LUSC, basal-like breast cancer—basal, head and neck squamous cell carcinoma—HNSC, lung adenocarcinoma—LUAD, ovarian carcinoma—OVCA, bladder urothelial cancer—BLCA, uterine corpus endometrial carcinoma—UCEC, and colon adenocarcinoma—COAD). Nineteen genes identified overlapped with the original lung cancer EMT signature, and when applied over 11 tumor types (those used to derive the signature, plus kidney clear cell carcinoma—KIRC, and rectal adenocarcinoma—READ), a wide range of the pan-cancer EMT signature gave a wide range of scores (Figure 1D). As expected, the pan-cancer signatures identify KIRC as highly mesenchymal and both READ and COAD as highly epithelial, in agreement with existing knowledge identifying these cancer types as such.

To better understand tumor gene expression pathways globally dysregulated in the context of EMT, we performed a pathway analysis of all genes correlated with the pan-cancer EMT score in all 11 tumor types. In addition to EMT pathways, among the top hits were pathways related to immune cell signaling. In the context of data generated by our group showing a relationship between EMT and immune escape [20], we investigated the relationship between the EMT score and expression of 20 potentially targetable immune checkpoint genes (Figure 1E). Across all the tumor types tested, we observed a strong positive correlation between EMT score and expression of the targetable immune checkpoint genes. This enrichment of immune target expression in mesenchymal tumors corroborated other work in our group in lung cancer where lung adenocarcinomas with a high lung cell line EMT score had high expression of PD-L1, which is a target of miR-200, which is also a suppressor of EMT and metastasis [20].

As a validation of the association between EMT and immune checkpoint genes, we stained lung adenocarcinoma sections included in a tissue microarray developed from the PROSPECT trial for expression of PD-L1. Automated quantification of immunohistochemistry (IHC) staining (H-score, calculated by multiplying extent and intensity of staining [21]) showed significantly higher expression of PD-L1 in both tumor and non-tumors cells in tumors with a mesenchymal pan-cancer EMT score (Figure 1F). As PD-L1 expression is a biomarker of response to PD-L1 blockade [22], by virtue of mesenchymal tumors expressing higher PD-L1, our analyses indicate that patients with mesenchymal tumors are more likely to be candidates for PD-L1 blockade, and other similar immune checkpoint blockade treatments.

The approach of using a “seed” to generate cell line and tumor-based signatures to quantify a biological program has been demonstrated in our work both to define alterations in signaling pathways and to identify therapeutic vulnerabilities. This signature generating approach has the potential to be applied to any scenario in which a few known markers describing two distinct morphologies or states have been defined.

### 3.2. Proteomic Subgrouping of SCLC

Proteomic profiling by RPPA measures a discrete number of targets enriched for druggable and oncologically important pathways (typically around 200 total/phosphorylated proteins) [23,24], and offers significant advantages over other profiling approaches. For example, proteomics, unlike DNA- or RNA-based profiling, directly measures pathway activation and candidate target expression (i.e., the protein “target” itself) [25]. Furthermore, protein biomarkers, particularly those that can be assayed by IHC have the potential for rapid translation into the clinic, as illustrated by the clinical use of PD-L1 IHC in NSCLC [22], and MET IHC in breast cancer [26]. 

**Figure 2:**
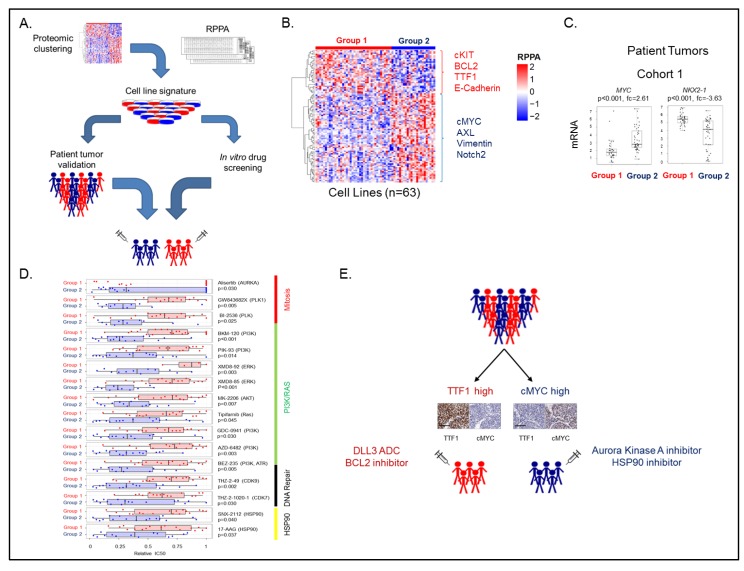
Proteomic subtyping of SCLC. Schematic of how SCLC was divided into two molecular subgroups using proteomic profiling data from 63 cell lines (**A**). Supervised hierarchical analysis shows distant protein expression patterns between the two cell line subgroups (**B**). Comparison of *MYC* and *NKX2-1* expression between the two subsets in patient tumors (**C**). Comparison of relative IC_50_ values between the two subsets shows group 2 (TTF1 low) to be more sensitive to a range of targeted agents (**D**). Working model of how SCLC patients may be divided into two groups (**E**) based on immunohistochemistry (IHC) tests currently in clinical use with different therapeutic vulnerabilities between the groups. Adapted from Cardnell et al 2017 [31]. RPPA—reverse phase protein array.

Clinically, SCLC is currently treated as a single disease, with all patients receiving essentially the same standard-of-care (SOC) treatment. The variability in response to SOC seen in the clinic, however, suggested a need to identify subgroups of SCLC with specific vulnerabilities that could be leveraged to develop more personalized approaches. Using proteomic data for 169 targets from a panel of 63 SCLC cell lines [27], we used a model-based clustering method [28,29] to determine the optimal number of clusters. Specifically, the cell lines were categorized into subgroups (range 1–20) using six distinct models, and Bayesian index clustering (BIC) was then applied to determine to optimal number of groups. The optimal model/group combination was then used to segregate the cell lines into two groups. When separated into two groups, we used two sample *t*-tests to compare expression of protein markers between the groups, identifying TTF1 and cMYC as the highest expressed proteins in groups 1 and 2, respectively (Figure 2B). Differences in expression of total protein between the cell line groups were then verified using publically available RNASeq data [30]. As cell culture may impact gene/protein expression, we used two cohorts of human SCLC tumors with gene expression data to validate our observations [7,8]. Using the 38 genes corresponding to total protein differences observed in the cell lines, we clustered the human samples. At the highest level, both patient cohorts separated into two groups, with striking differences in *NKX2-1* (the gene name of TTF1) and *MYC* between the groups. 

**Figure 3:**
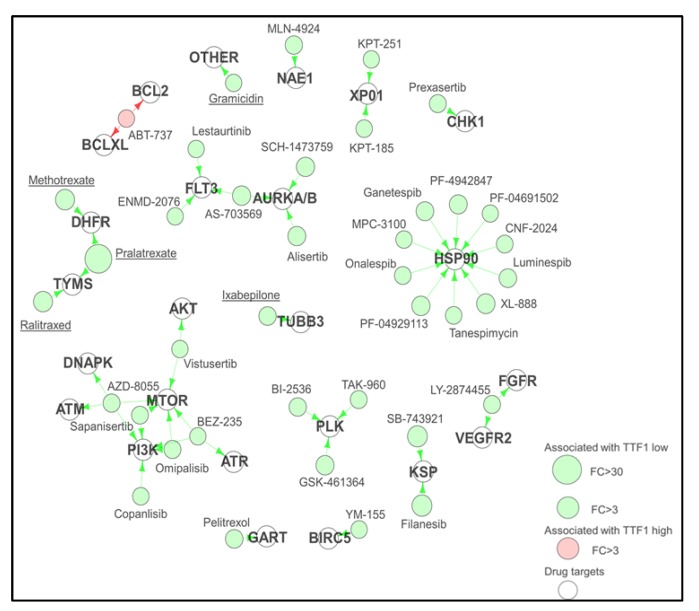
Drug-target constellation (DTECT) mapping. DTECT map of drugs differentially sensitive between TTF1 high and low SCLC cell line (Fold Change (FC) > 3.0, *p* < 0.001). Drugs with differential sensitivity are mapped by their primary, secondary, and tertiary targets. Underlined drugs are either FDA approved or licensed for use in Canada/Europe. Adapted from Cardnell et al. 2017 [31].

Having identified two proteomically defined subsets of SCLC, we used a combination of internal [27] and publically available drug sensitivity data [30,32] to determine if these groupings drove differential responses to candidate treatments. Having identified a large number of targeted agents with differential sensitivity between the two subgroups of SCLC, we were intrigued to see if we could use information about the drug targets to identify targets common to multiple drugs. Adapting our clustering data, as expression of TTF1 is bimodal, we segregated the cell lines into two groups (TTF1 high and low) and identified drugs that had a minimum three-fold difference in mean IC_50_ between TTF1 high and low cell lines. We then used an in-house curated drug target database that includes the primary, secondary, and tertiary targets of a given agent to generate a “Drug-TargEt ConsTellation map” (DTECT map—Figure 3). The DTECT map identified multiple common targets including Aurora Kinase and the PI3K/mTOR pathway, similar to the group-based analysis (Figure 2), confirming the validity of the approach. DTECT mapping is an approach that can be used to identify high priority drug targets in any situation where cell lines can be dichotomized on the basis of gene or protein expression, or other statuses such as epithelial versus mesenchymal. The Aurora Kinase inhibitor alisertib has shown pre-clinical and clinical activity in a number of cancer types, including SCLC [33,34]. However, in a phase II study of paclitaxel with alisertib or placebo in an unselected relapsed/refractory SCLC cohort, treatment with alisertib did not improve response rates or survival [35]. As our sub-group comparisons, as well as DTECT mapping using both TTF1 and cMYC expression, had all indicated that Aurora Kinase inhibition was more effective in cMYC high SCLC cell lines, we performed a supervised analysis of candidate proteomic biomarkers of response to single agent alisertib in a panel of 51 SCLC cell lines [31]. Using two approaches (correlating IC_50_ values to protein expression, and comparing protein expression between the most and least sensitive models), high cMYC expression was the top biomarker of sensitivity to alisertib. A retrospective analysis of biopsies from patients enrolled in the phase II trial of alisertib, based on our preliminary data, showed a strong association between cMYC protein expression and improved progression free survival, validating our pre-clinical analyses, despite only being evaluable in a small number of patients [35]. Alisertib is no longer in clinical development for SCLC, in large part because of the lack of efficacy in the unselected phase II trial. An alternative study in an SCLC population selected for patients with tumors that express high levels of cMYC or low TTF1 (for which CLIA certified assays are available) may have yielded a positive outcome and highlights the utility of high-throughput biomarker discovery in the development and subsequent use of novel therapeutics.

### 3.3. DISARM

**Figure 4:**
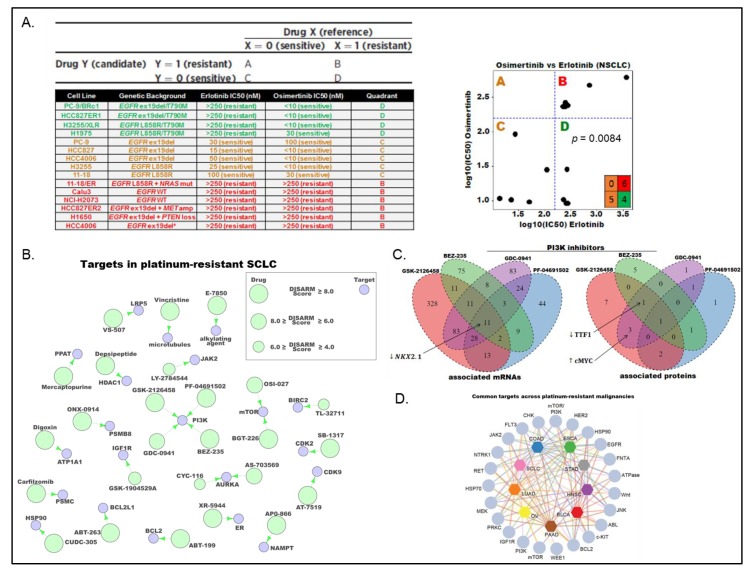
DISARM. DISARM places cell lines, based on IC_50_ data, into a 2 × 2 grid. Using data for erlotinib (reference) and osimertinib (candidate), DISARM correctly identifies osimertinib as a candidate drug in erlotinib-resistant *EGFR T970M* mutant NSCLC (**A**). DTECT mapping of top candidate drugs identified by DISARM in cisplatin-resistant SCLC (**B**). Venn diagrams depicting shared mRNA and protein biomarkers of sensitivity to multiple PI3K inhibitors in platinum-resistant SCLC (**C**). Map demonstrating the interrelatedness of drug targets identified as candidate drugs for cisplatin-resistant disease across multiple cancer types (**D**). Adapted from Gay et al. 2018 [39].

Over recent years, a tremendous quantity of publically available drug-sensitivity data has been generated using a plethora of therapeutic agents across multiple cancer types [30,32,36,37,38]. Our tools and approaches to utilizing this data have, however, not developed at the same rate. Pre-clinical data generated in unselected populations may result in potential therapies being discarded because of their lack of efficacy in the overall population despite their potential efficacy in a targeted population (e.g., Aurora Kinase inhibitors in MYC high SCLC). Similarly, answering the supposedly simple question of, “if a group of tumor models are resistant to a given drug, to what are they sensitive?” is not easily addressed and is often unintentionally biased by the researchers’ pre-existing knowledge. To address this, we developed DISARM (Differential Sensitivity Analysis for Resistant Malignancies), a bioinformatics tool designed to identify drugs with efficacy in models that are resistant to a reference drug [39]. DISARM operates by comparing IC_50_ values for two drugs (the reference and candidate drugs), placing them into a 2 × 2 table to identify instances in which a significant number of models are sensitive to a candidate drug and are resistant to the reference drug (Figure 4A). DISARM calculates a score—the DISARM score—for each drug combination that follows a standard distribution. A higher DISARM score corresponds to a higher significance level, the minimum score is zero and, while there is no theoretical maximum to the score, a score of ≥2 is considered to be meaningful. This approach was validated using two clinical paradigms where there is an approved treatment option (candidate drug) for patients with tumors resistant to standard of care (reference drug). One such scenario is the treatment of metastatic NSCLC with exon 19 deletions or *L858R* mutations in *EGFR*. Here, SOC therapy includes the tyrosine kinase (TKI) inhibitor erlotinib (EGFR inhibitor), to which resistance invariably develops, approximately half of which occurs through the acquisition of an additional *T790M* mutation in *EGFR*. A second generation EGFR inhibitor—osimertinib—is, however, effective in patients with *EGFR T970M* erlotinib-resistance mutations. Using response values to both erlotinib and osimertinib from a previous study [40], DISARM successfully identified osimertinib as a candidate for cell lines with *T970M EGFR* mutations (Figure 4A). 

We subsequently used DISARM to interrogate data from a large NCI funded drug screen effort in SCLC [30]. Using sensitivity to platinum, the backbone to all frontline SOC treatments for SCLC [41] to which resistance develops rapidly and almost universally, as the reference drug, we applied DISARM to the problem of platinum-resistance in SCLC. Using cisplatin sensitivity data from our laboratory and data from 526 FDA approved an investigational anti-cancer agent [30], DISARM selected 31 candidate drugs (including 26 with defined molecular targets) for use in platinum-resistant SCLC. The 26 candidate drugs with defined molecular targets, all of which had DISARM scores ≥4.0 were then plotted using a DTECT map of their primary target, which revealed a number of common targets including PI3K, mTOR, and Aurora Kinase A (Figure 4B). We then tested if cell lines identified by DISARM on the basis of their sensitivity to a candidate drugs targeting the same molecule shared common biomarkers of sensitivity. Comparing mRNA [30] and protein expression data [27,42] between cell lines identified as sensitive and resistant by DISARM, we identified low expression of the gene *NKX2-1* and its protein (TTF1) as common markers of sensitivity to PI3K inhibitors (Figure 4C), in agreement with our proteomic subtyping of SCLC (Figure 2D) [31].

As platinum-resistance is not unique to SCLC, we expanded our analysis to include nine solid tumor types for which platinum-based therapy is an established frontline therapy according to National Comprehensive Cancer Network (NCCN) guidelines [43]. These included SCLC, NSCLC, stomach adenocarcinoma (STAD), pancreatic adenocarcinoma (PAAD), ovarian (OV), head and neck squamous cell carcinoma (HSC), esophageal carcinoma (ESCA), colon adenocarcinoma (COAD), and bladder carcinoma (BLCA). Using IC_50_ data for 138 drugs for which there were adequate data across these nine tumor types in the GDSC database [32], we used DISARM to identify common drugs and drug targets across cisplatin-resistant models of different tumor types (Figure 4D). Although sensitivity to many candidate drugs varies between cisplatin-sensitive and -resistant disease for many tumor types, some patterns did emerge. For example, vinblastine and etoposide consistently performed better in cisplatin-resistant models, with DISARM scores of ≥2 in 4/7 and 5/7 tumor types tested, respectively. DISARM analyses also revealed a number of common drug targets across multiple platinum-resistant malignancies including PI3K, mTOR, MEK, BCL-2, and HSP-90.

In order to make DISARM available to the broader cancer research community, we also developed a Java-Script based webtool to all investigators to query the available databases with a disease, reference drug, and cut-offs for sensitivity of their choice. The DISARM web-based tool is available at http://ibl.mdanderson.org/DISARM/index. The analyses presented here and in the DISARM manuscript are only the beginning of how this approach can be used to interrogate in vitro data from datasets that are yet to be incorporated, such as the Connectivity Map [44] and from individual investigators. While not yet explored in our analyses, DISARM has the potential to be applied beyond the setting of in vitro drug response data. If properly adapted and validated, DISARM-like approaches could be applied in the analysis of in vivo drug response data using parameters such as tumor volume or ΔT/ΔC in lieu of IC_50_ values. Taking the concept further, scenarios in which DISARM could be used in the analysis of clinical data combining together tumor types with shared drug resistance can also be envisioned.

## 4. Conclusions

The approaches reviewed here represent the work of a large multi-disciplinary team that utilized large datasets to develop approaches for the unbiased classification of models/tumors and identification of novel candidate drugs. These approaches have incorporated both large cell line datasets (proteomic profiling, transcriptomic data, genomic data, and drug sensitivity data) from multiple sources along with patient derived data (transcriptomic, genomic, immunohistochemical, and clinical) from large collaborative efforts (TCGA), publically available data (George et al., Sato et al.; [7,8]), as well as clinical trials (BATTLE-1, BATTLE-2, PROSPECT; [18,21,45]) from multiple cancer types. One example of how these and related approaches have altered our approach to the treatment of lung cancer patients is the discovery of SLFN11 as a biomarker of response to PARP inhibition in SCLC. A proteomic comparison of NSCLC and SCLC led to the initial discovery of PARP1 as a potential therapeutic target in SCLC [42], which was validated in vitro, in vivo, and in SCLC patients [46,47]. Further biomarker analysis using proteomic and transcriptomic profiling data in combination with response data to PARP inhibition in cell lines and PDX models led to the identification of SLFN11 as a biomarker of response [48]. Subsequent retrospective analysis of biopsies from patients enrolled in a Phase II study of temozolomide with or without veliparib (a PARP inhibitor) showed a survival advantage for patients with SLFN11 positive tumors (by IHC) who received veliparib [49].

The tools developed by the University of Texas MD Anderson Lung Cancer Multidisciplinary Program have been highly productive for advancing our understanding of both thoracic and extra-thoracic cancers, particular in expanding our knowledge of the mechanisms of resistance to treatment and identifying new treatment options for patients for whom no treatment options currently exist. These tools, used in the context of multi-disciplinary teams, have the potential to be further leveraged to explore a variety of questions about the biology of lung and other cancers, but most importantly, have the potential to translate into novel, biomarker-driven, personalized treatments for our patients.

## Figures and Tables

**Table 1:** Publically available datasets. Summary of publically available datasets used in the approaches presented. * Datasets obtained from the National Center for Biotechnology Information (NCBI) for these analyses include those from George et al., Sato et al. [7,8], BATTLE-1, BATTLE-2, and PROSPECT. # Data types available vary by study. TCGA—The Cancer Genome Atlas; CCLE—Broad Institute Cancer Cell Line Encyclopedia; GDSC—Genomics of Drug Sensitivity in Cancer; SCLC—small cell lung cancer; EMT—epithelial-to-mesenchymal transition.

Resource	Malignancy	Data Types	Pre-Clinical/Clinical	Approach
TCGA [9]	Various	Genomic, transcriptomic, methylation, copy number, proteomic, and clinical #	Clinical	EMT
NCBI * [10]	Various	Genomic, transcriptomic, methylation, copy number and clinical #	Both	EMT, SCLC subgroups
CCLE [11]	Various	Drug sensitivity, genomic, and transcriptomic	Pre-clinical	SCLC subgroups, DISARM
GDSC [12]	Various	Drug sensitivity, genomic, and transcriptomic	Pre-clinical	SCLC subgroups, DISARM
NCI Developmental Therapeutics Program [13]	SCLC	Drug sensitivity, and transcriptomic	Pre-clinical	SCLC subgroups, DISARM
DISARM [14]	Various	Drug sensitivity	Pre-clinical	DISARM
